# Both acute and chronic caffeine consumption affect cardiovascular responses to total sleep deprivation

**DOI:** 10.3389/fcvm.2026.1718154

**Published:** 2026-01-30

**Authors:** Lise Mateo, Pierre-Emmanuel Tardo-Dino, Danielle Gomez-Merino, Catherine Drogou, Pierre-Emmanuel Josse, Mégane Erblang, Philipe Colin, Marie Claire Erkel, Pascal Van Beers, Damien Leger, Cyprien Bourrilhon, Mounir Chennaoui, Fabien Sauvet

**Affiliations:** 1 Faculté de Médecine et de Sciences de la Santé, Université de Bretagne Occidentale (UBO), Brest, France; 2 Académie de Santé des Armées, Ecole du Val de Grâce, Paris, France; 3 Institut de Recherche Biomédicale des Armées (IRBA), Brétigny sur Orge, France; 4 UMR LBEPS, Univ Evry, IRBA, Université Paris Saclay, Evry, France; 5 Université Paris Cité, VIFASOM, (UMR Vigilance Fatigue Sommeil et Santé Publique), Paris, France; 6 APHP, Hôtel-Dieu, Centre du Sommeil et de la Vigilance, Centre de Référence Narcolepsies et Hypersomnies Rares, Centre de Ressources en Pathologies Professionnelles et Environnementales ‘Sommeil Vigilance et Travail’, Paris, France

**Keywords:** blood pressure, caffeine, cardiovascular risk, inflammation, recovery, sleep deprivation

## Abstract

**Introduction:**

Sleep deprivation is known to induce cardiovascular responses. Several studies have shown the beneficial effect of caffeine on neurobehavioral performance during sleep deprivation, but less is known about its influence on the cardiovascular and inflammatory responses associated with sleep deprivation. The aim of this study is to evaluate the impact of acute caffeine intake on (1) peripheral blood pressures, and (2) heart rate, and cutaneous vascular conductance (CVC) and related biomarkers of endothelial activation, during total sleep deprivation (TSD), considering habitual caffeine consumption.

**Methods:**

41 subjects followed a randomized, placebo-controlled, cross-over study and underwent 2 conditions of TSD (38 h), one with caffeine intake at 09:00 and 14:00 (2.5 mg/kg), and the other with placebo intake.

**Results:**

We confirm that TSD increases systolic and diastolic arterial pressures (*p* = 0.001 and *p* = 0.002 for main effects respectively) and heart rate (*p* = 0.001), and decreases endothelium-dependent and -independent CVC (*p* = 0.001). Acute caffeine intake inflates the increase in arterial pressures and IL-6 levels, while it does not affect CVC and levels of E-selectin and monocyte chemotaxis protein-1 (MCP-1). Moreover, chronic caffeine consumption had significant main effects on systolic arterial pressure (*p* = 0.03), heart rate (*p* = 0.02), IL-6 levels (*p* = 0.02), and acetylcholine (ACh)-induced CVC (*p* = 0.02), and interacted with TSD on E-selectin levels and ACh-induced CVC (*p* = 0.02 respectively).

**Conclusion:**

Acute caffeine intake provokes immuno-inflammatory and cardiovascular responses, and chronic caffeine consumption should be limited to the lowest efficient doses.

**Clinical Trial Registration:**

https://clinicaltrials.gov/study/NCT03859882, identifier NCT03859882.

## 1 Introduction

Sleep is a period of inactivity and rest essential for homeostasis and health. However, 16.3% of active workers in France are night workers who are repeatedly exposed to total sleep deprivation (1). Many sectors are affected, among them military personnel, health professionals, security officers, or even truck drivers. In addition to these total sleep deprivations, there is a reduction in overall sleep time. According to a 2019 study of 12,637 participants in France, the average total sleep time of French people is less than 7 h of sleep. More than 35% of the population in this study was in sleep debt (2). Due to habits such as screen use (telephone and computer), night work and caffeine consumption, more than a third of people sleep too little or not at all (2). Moreover, a single night of total sleep deprivation is sufficient to induce a degradation of attentional, cognitive and physical performances (3–5) and to induce changes in neuroendocrine, metabolic, inflammatory, and cardiovascular responses (6–8). When sleep debt becomes chronic, there is a tendency to increase the risk of cardiovascular and metabolic diseases (9, 10).

Caffeine is one of the main countermeasures used to partially restore endurance and strength performance, as well as to reduce the feeling of stress and fatigue (11–13). Caffeine consumption has doubled in the world in 20 years (14). To some extent, it helps prevent cognitive performance impairment caused by sleep deprivation (15, 16). Due to these beneficial effects, caffeine is commonly consumed by personnel in atypical or shift work environments such as night shift workers, healthcare professionals, or serving military personnel (17). A 2012 observational study described that 82% of active U.S. military personnel consume coffee or caffeine-containing products (17), which, at large doses, could potentially aggravate or contribute to the development of certain cardiovascular diseases in some hypertensive or hypertension-prone subjects (18).

Despite its beneficial effects, caffeine consumption is not free of side effects and health risks. Indeed, the main side effects identified in the literature and associated with this molecule are anxiety, sleep disorders, tachycardia, nausea… (19). According to some authors, there is also a risk factor for cardiovascular and osteoporosis, or even pregnancy complications (19). We also recently showed that chronic low-caffeine consumers are faster on attentional performance during sleep deprivation than moderate- and high-caffeine consumers (20). As a result, many authors recommend limiting its use (19, 21). It therefore appears that, while the cognitive benefits of caffeine are well known, few studies have explored its effects on cardiovascular disease and its development, particularly in cases of acute total sleep deprivation. Moreover, studies on the beneficial cognitive effects of caffeine under sleep deprivation have often been conducted after a period of caffeine withdrawal (15). It therefore seems interesting to study the risk/benefit balance of acute caffeine intake on cardiovascular responses in healthy subjects deprived of sleep and not withdrawn from caffeine to remain as close as possible to everyday life conditions.

The objective of this laboratory study was to evaluate the impact of acute caffeine intake on cardiovascular parameters during a total sleep deprivation protocol (38 h of continuous wakefulness) in healthy subjects, taking into account habitual caffeine consumption, and without caffeine withdrawal. The primary endpoint was an average of three self-measurements of systolic and diastolic blood pressure, assessed every 6 h. Secondary objectives were to assess heart rate, endothelial function, and immunoinflammatory responses.

## 2 Materials and methods

### 2.1 Participants

Healthy participants, aged between 18 and 55 years, were included. The study (N° IDRCB ID-RCB: 2017-A02793-50) received the agreement of the Cochin—CPP Ile de France IV (Paris) Ethics Committee and of the French National Agency for Medicines and Health Products Safety (ANSM) (Ile de France IV) and was conducted according to the principles expressed in the Declaration of Helsinki of 1975, as revised in 2001. All the participants gave their informed written consent. This study is a part of the PERCAF clinical trial (NCT03859882).

Participants were free from medical, psychiatric, and sleep disorders. Other exclusion criteria included physical or mental health disorders based on (I) Hospital Anxiety and Depression scale, HAD ≥ 16 (22), (II) significant medical history, (III) Epworth Sleepiness Scale, ESS > 10 (23), (IV) Pittsburg sleep quality index, PSQI > 8 (24), (V) morningness-eveningness questionnaire, <31 or >69 (25), (VI) habitual time in bed per night <6 h (16, 26). We also excluded subjects younger than 18 years and older than 55 years, with a body mass index (BMI) greater than 30 kg/m^2^, working at night or in shift work or jet lag (>3 time zones) in the previous month, drinking more than 1 glass of alcohol per day, and abusing tobacco. Habitual coffee consumption (number of cups per day), smoking habits, weekly consumption of alcohol and time physical activity were assessed with a questionnaire (27). Participants completed a sleep/wake schedule and maintain their sleep and caffeine consumption patterns for the week prior to the study. After the selection between September 2018 and January 2019, subjects were definitively enrolled in the experimental design between March and June 2019.

### 2.2 Study design and testing conditions

This randomized crossover study has been conducted in the sleep laboratory of the Armed forces biomedical research institute (IRBA), Brétigny sur Orge, France. Ambient temperature was controlled and maintained at 22 °C (± 1 °C) during all the experiment. The brightness of the lighting has been maintained between 150 and 200 lux during the awaking periods and lights were off during sleep periods. Meals and caloric intake were standardized for a caloric intake not exceeding 2,600 kcal/day. Mealtimes were fixed (08:15–08:45 for breakfast, 12:15–13:00 for lunch and 19:00–20:00 for diner). A snack was provided between 01:00 and 02:00. At dinnertime, the subjects took their showers.

**Figure 1:**
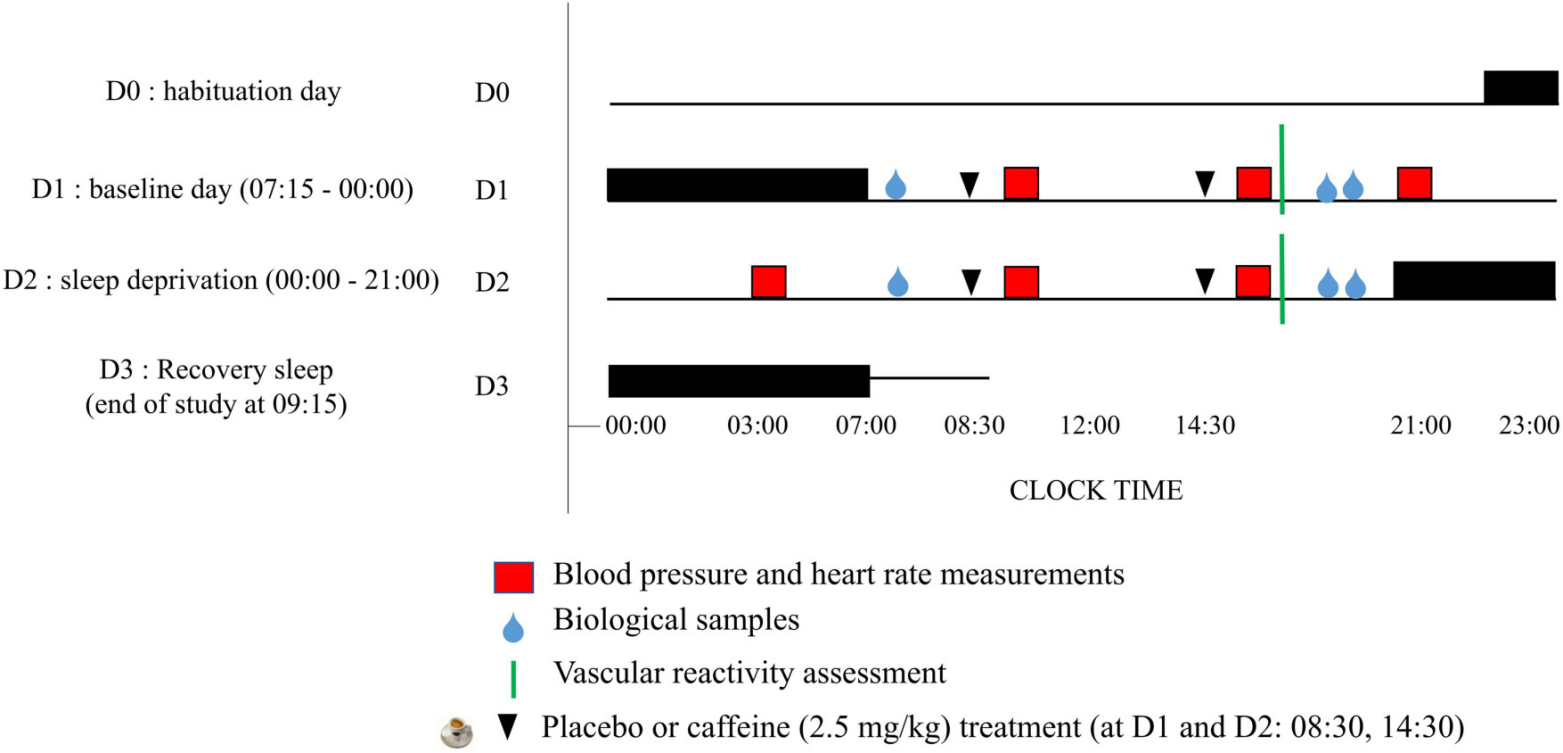
Experimental design. D0 is the habituation day, D1 is the baseline day, D2 is the day of prolonged wakefulness (i.e., sleep deprivation, between 00:00–21:00) and D3 is the recovery sleep and end of the study. Night sleep are the horizontal black rectangles, and caffeine or placebo intake at 08:30 and 14:30 are the black arrows.

The subjects were accommodated in the laboratory's special “sleep” apartment for 3 consecutive days. The experimental protocol included (I) a habituation/training day (D0) where subjects went to bed at 22:00, (II) a baseline day (D1) beginning at 07:15 (wake time) until 00:00, (III) a total sleep deprivation (TSD) day beginning on D2 00:00 until 21:00 (i.e, 38 h of continuous wakefulness), and (IV) a recovery night until the end of the study (09:00 on D3) (Figure 1). Participants were welcomed in groups of 4 around 16:00 on D0 day. During this laboratory protocol, participants were always under visual surveillance of research staff members. Activities such as meals, showers and leisure activities, were only possible at specific controlled times.

When participants were not engaged in testing, meals, or sleep periods, they were not allowed to exercise, use tobacco, alcohol, or other psychoactive substances. However, they were allowed to read, to watch videos, or to speak with other participants or staff members and playing games. In addition, participants wore a wrist actigraphy to check that they stayed awake during the 38-h continuous wakefulness period.

### 2.3 Caffeine administration

In this double-blind, crossover study, participants followed two conditions (i.e., caffeine or placebo, administered twice on D1 and D2), with a 2-week washout period between the two conditions during which they returned to their off-protocol lifestyle. Each participant in the caffeine group received 2.5 mg/kg of body weight of caffeine in the form of a powder mixed with a decaffeinated beverage. Caffeine intake (amount and interval between doses) followed McLellan's (13) recommendations for improving attention in sleep-deprived individuals, which themselves took into account the metabolism and kinetics of caffeine's effects.

Placebo was a decaffeinated beverage with the same bitterness, smell, and taste. The caffeine powders were pre-measured by the project supervisor. This amount of caffeine powder was chosen for its enhancing properties on attention in sleep-deprived conditions (2.5–8 mg/kg of caffeine) (13). The beverage was administered at 08:30 and 14:30 (i.e., at 1.5 and 7.5 h of prolonged wakefulness) on D1, and at 08:30 and 14:30 (i.e., at 25.5 and 31.5 h of prolonged wakefulness) on D2.

### 2.4 Evaluation of cardiovascular parameters

#### 2.4.1 Blood pressure and heart rate assessment

Blood pressure and heart rate were self-measured using an automatic blood pressure monitor (M10-IT, HEM-7080IT-E, OMRON). Three measurements were made with a 30 s interval between each measurement. This method has been used for the oscillometric blood pressure monitoring device validation according to the British Hypertension Society protocol (56) and in precedent studies (56).

This evaluation was carried out every 6 h, after a period of questionnaires and 30-minute cognitive tests carried out in a sitting position. It was performed at 09:00, 15:00 and 21:00 on day D1, and at 03:00, 09:00 and 15:00 on day D2 respectively, corresponding to 2, 8, 14, 20, 26, and 32 h of continuous wakefulness. The subjects were previously trained to perform this measurement. The position of the arm and armband was displayed in front of them. This choice of self-measurement was justified by the number of subjects (4 patients at the same time), as well as to avoid overestimation of blood pressure by the white coat effect. This self-measurement is a reference for the measurement of ambulatory blood pressure and is recommended by the French National Authority for Health (HAS) for the diagnosis of hypertension (28–30).

#### 2.4.2 Vascular reactivity assessment

**Figure 2:**
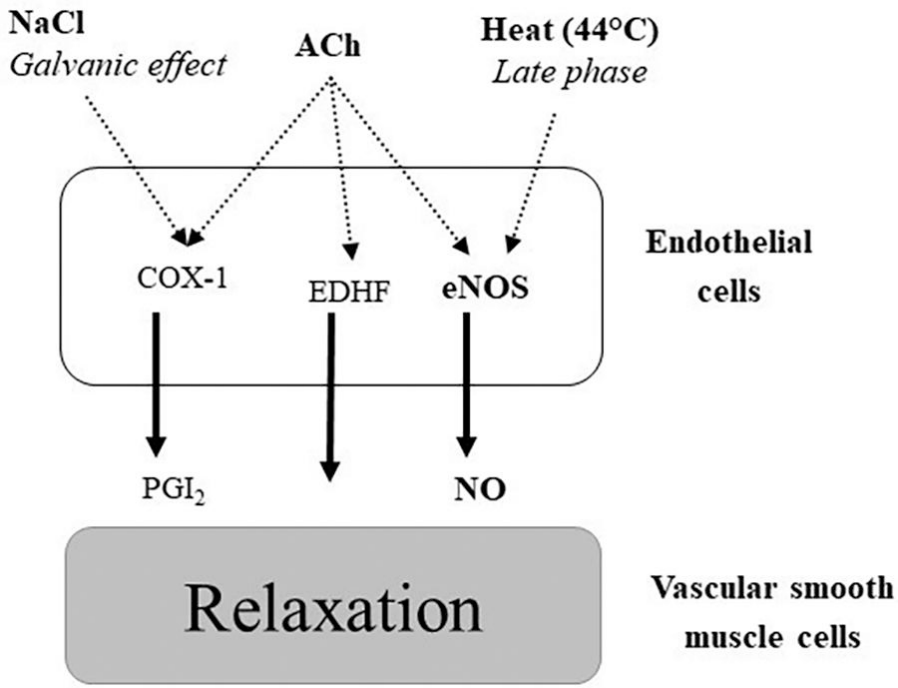
Pathways implicated in vasodilation after local application of acetylcholine (ACh), NaCl and heat (44 °C). COX-1: cyclooxygenase 1, PGI2: prostaglandin I2 (prostacyclin), EDHF: endothelium-derived hyperpolarizing factors, eNOS: endothelial nitric oxide synthase.

Vascular reactivity was estimated at 14:00, by measuring the increase in cutaneous blood flow after local application of acetylcholine (ACh), NaCl and heat (44 °C) using a doppler laser on the arm (Figure 2). These measures are perfectly painless and without side effects (7). Skin blood flow (SkBF) was measured by three Doppler laser flow meter probes (481-1, Perimed; Stockholm, Sweden), connected to a laser Doppler unit (PF5000, Perimed).

Cutaneous vascular conductance (CVC, expressed as PU/mmHg) is calculated as the ratio of SkBF to mean arterial pressure (MAP) measured every 10 min during the experiment. The baseline values are obtained by averaging the values of the last two minutes of the five-minute rest period prior to the iontophoresis of ACh, NaCl and heat application. Data collection was recorded continuously for 30 min. Finally, all results are expressed as a percentage of the baseline CVC value (% baseline). The CVCpeak is defined as the maximum value averaged over 30 s, recorded after the start of heat application and iontophoresis.

Cutaneous vasodilation induced by small doses of ACh, NaCl or local heat application are used as quality markers of endothelial function. Their decrease is considered an index of endothelial dysfunction, which in turn is associated with increased cardiovascular risk (31, 32). Endothelial dysfunction is associated with deterioration of endothelial-dependent vasodilatation, mainly caused by the alteration of NO bioavailability and is a marker of cardiovascular risk (33–35).

#### 2.4.3 Endocrine and immuno-inflammatory response

In accordance with the experimental protocol, blood samples were taken after 1 h (i.e., at 8 a.m., corresponding to baseline), 11, 13, 25, 35, and 37 h of continuous wakefulness. Measurements of plasma or serum levels of IL-6, E-selectin, and monocyte chemoattractant protein-1 (MCP-1) were performed. These measurements are carried out using commercially ELISA kits (R&D Systems; Minneapolis, MN). A total of 3.6 mL was taken at each blood test, for a total of 144 mL of blood. The tubes were immediately centrifuged after sampling and plasma and serum aliquots were immediately frozen at −80 °C for further data analysis.

IL-6 has been chosen because it increases with sleep deprivation (7, 36) and caffeine intake associated with exercise (37), and is implicated in the vascular function and cardiovascular risk (7). Furthermore, changes in the concentration of the cytokine IL-6 (both pro- and anti-inflammatory) linked to physiological stress (sleep deprivation or exercise) in healthy individuals have been fairly easy to quantify for several years. In contrasts, for example, with the quantification of the two pro-inflammatory cytokines TNF-α and IL-1β. E-selectin and MCP-1 are biological index of endothelial dysfunction (7, 34, 38).

### 2.5 Statistics

Statistical analyses were carried out with the JAMOVI software for R. The significance threshold was set at 5% (alpha risk).

For the main judgement criterion, namely blood pressure (systolic, diastolic, and mean), given the scientific literature and the results observed in our previous work (7, 8, 38), 12 subjects per group are needed to show a sleep deprivation effect. This number was calculated taking into account the variability observed (18%) in our studies, the alpha risk of 5%, a power of 80% and a desired difference of 30% between values. This number is adapted to repeated measurements, with each subject serving as their own control.

We used a mixed linear model (function lmr) with a fixed factor in repeated measurement (treatment: caffeine vs. placebo), a linear factor in repeated measurement (wake time, 1 point/6 h) and a quantitative factor without repeated measurement, chronic caffeine consumption. The repeated measurement was obtained by adding a random effect to the model for the subjects for treatment and wake time. The same analysis was conducted for heart rate, biomarkers, and vascular reactivity responses. In case of significant interactions, mean values were compared using a Bonferroni *post-hoc* test.

Secondarily, subjects were clustered (divided) into 3 groups based on their habitual daily caffeine consumption: low (<50 mg/day), moderate (50–300 mg/day) and high (>300 mg/day) (20). When an effect of habitual consumption was observed, the parameters were represented according to the 3 groups and the mean values compared using a *post-hoc* Bonferroni test.

Finally, correlations between the values were carried out using Pearson analysis on the raw data but also on the delta [(D2-D1)/D1].

## 3 Results

### 3.1 Participants

42 subjects were enrolled in the experimental design. We excluded one participant because of an important adverse effect after caffeine intake. Finally, a total of 41 healthy participants (33.2 ± 0.9 years) fully followed the protocol, including 53.7% (*N* = 22) of women and 43.6% (*N* = 19) of men.

Participants' average daily caffeine consumption was 247 ± 23 mg (mean ± SEM). The low caffeine consumers consumed 14.0 ± 18.8 mg/d (*n* = 11), the moderate consumers 194 ± 66 mg/d (*n* = 12), and the high consumers 431 ± 108 mg/d (*n* = 18).

Most participants were non-smokers or smoked fewer than 12 cigarettes per day. The mean weekly alcohol consumption was 1.9 ± 0.2 glasses. The mean BMI was 22.7 ± 0.6 kg/m^2^ for women and 23.3 ± 0.7 kg/m^2^ for men. The mean weekly exercise duration was 3.1 ± 0.3 h. The mean daily total sleep time was 7.3 ± 0.1 h and the sleepiness score was 6.8 ± 0.4. Chronotype was distributed this way: 35% had morning chronotype, 35% evening chronotype and 30% middle tier. Finally, no serious adverse effects on health (nausea, vomiting, abdominal pain, headache, fatigue) due either to sleep deprivation or to the caffeine/placebo treatment have been reported.

### 3.2 Systolic arterial pressure (primary endpoint)

**Figure 3:**
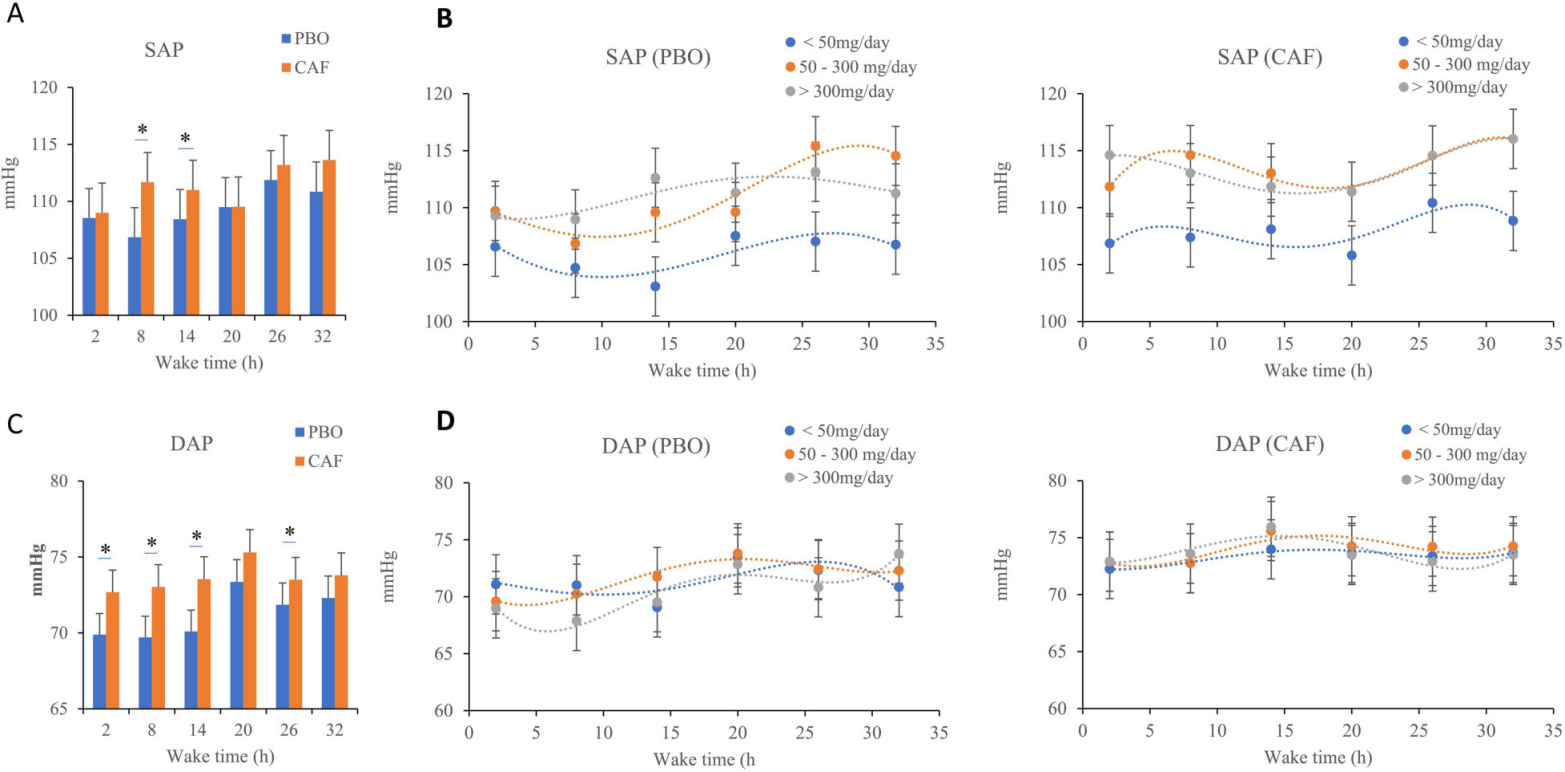
Mean systolic (SAP, **A, B** panels) and diastolic arterial pressures (DAP, **C, D** panels), measured every 6 h (2, 8, 14, 20, 26, and 32 h) during continuous wakefulness. **(B)** and **(D)** subfigures are acute placebo condition (PBO, on the left) and caffeine condition (CAF, on the right) according to chronic caffeine consumption groups [<50 mg/day: low consumer group (*n* = 11); 50–300 mg/day: moderate consumer group (*n* = 12); >300 mg/day: high consumer group, n = 18]. *p < 0.05, between PBO and CAF conditions.

We observed an effect of sleep debt (*p* = 0.001), acute caffeine intake (*p* = 0.01) and habitual caffeine consumption (*p* = 0.03), with no significant interaction (Table 1). A progressive increase in systolic arterial pressure (SAP) during sleep deprivation in both conditions is observed (Figure 3A). The SAP was higher in the caffeine group compared to the placebo group, and the measurements show higher SAP at 09:00 on the second day (26 h of continuous awakening) compared to the first day at the same time (Figure 3A). Subjects with high habitual caffeine consumption (>300 mg/day) had higher SAP than low consumers (<50 mg/day) in both the placebo and caffeine conditions during total sleep deprivation (Figure 3B).

**Table 1:** Results of mixed linear analyses for systolic arterial pressure (SAP), mean arterial pressure (MAP), diastolic arterial pressure (DAP) and heart rate (HR).

Mixed linear model effects	SAP	MAP	DAP	HR
Treatment (acute caffeine intake—TRT)	**7.11** **(0.01)**	2.31 (0.13)	**28.31** **(0.001)**	2.31 (0.21)
*F*_1,40_, (p)				
Wake time (TSD)	**7.11** **(0.001)**	**83.67** **(0.001)**	**5.39** **(0.002)**	**28.32** **(0.001)**
*F*_5,240_, (p)				
Chronic caffeine consumption (CC)	**2.72** **(0.03)**	0.81 (0.61)	1.07 (0.32)	**2.15** **(0.02)**
*F*_2,39_, (p)				
TRT × TSD	1.09 (0.38)	0.96 (0.48)	0.15 (0.97)	2.70 (0.10)
*F*_5, 387_, (p)				
TRT × CC	2.61 (0.07)	3.21 (0.07)	3.35 (0.07)	1.31 (0.65)
*F*_2, 40_, (p)				
TSD × CC	0.02 (0.95)	0.77 (0.63)	0.16 (0.69)	2.70 (0.83)
*F*_10,387_, (p)				
TRT × TSD × CC	0.79 (0.54)	0.59 (0.79)	0.07 (0.79)	0.11 (0.99)
*F*_10,387_, (p)				

Bold values are significant values.

### 3.3 Diastolic arterial pressure

The wake time and acute caffeine intake have significant effects on diastolic arterial pressure (DAP) (*p* = 0.002 and *p* = 0.001 respectively) (Table 1), without significant effects of chronic caffeine consumption (*p* = 0.32), and without significant interactions between chronic caffeine consumption, acute caffeine treatment, and sleep deprivation (*p* < 0.05). On Figure 3C, we see, as with SAP, an increase in DAP as sleep deprivation occurs, whether in the placebo condition or the caffeinated condition. There is a significant difference in DAP between the caffeine and the placebo conditions on the first day (at 2 h, 8 h and 14 h of continuous awakening, marked on the graph by the stars, Figure 3C). In both conditions of the study, the DAPs of the caffeine consumer groups follow the same curve (Figure 3D).

### 3.4 Heart rate (HR)

**Figure 4:**
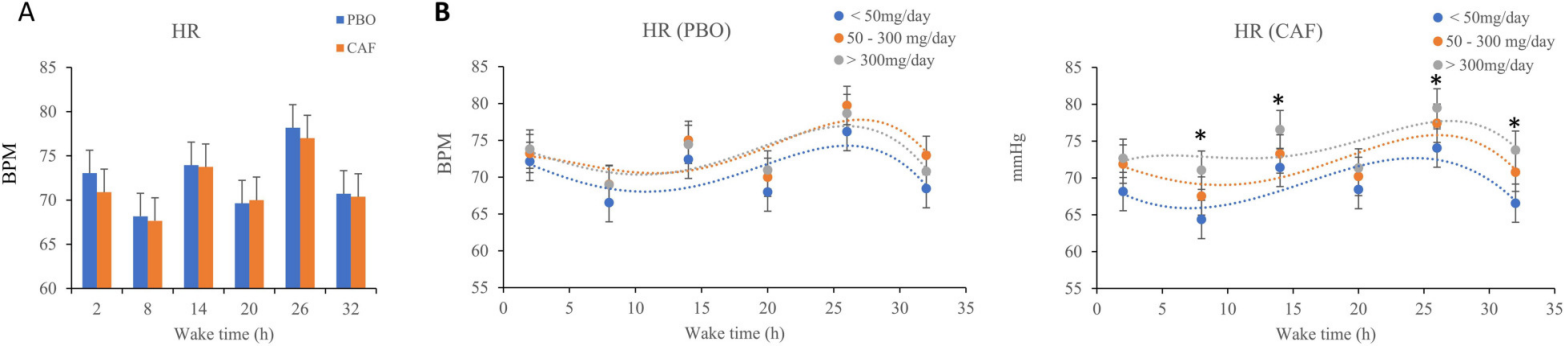
**(A)** Mean heart rate (HR), measured every 6 h (2, 8, 14, 20, 26, and 32 h) during continuous wakefulness, **(B)**. Values for acute placebo condition (PBO, on the left) and caffeine condition (CAF, on the right) conditions, according to chronic caffeine consumption [<50 mg/day: low consumer group (*n* = 11); 50–300 mg/day: moderate consumer group (*n* = 12); >300 mg/day: high consumer group, *n* = 18].

The wake time and chronic caffeine consumption have significant effects on HR (*p* = 0.001 and *p* = 0.02 respectively), with no significant effect of acute caffeine treatment and no interaction (Table 1). In the two conditions (placebo and caffeine), an increase in HR after 26 h of continuous awakening is observed (at time point 09:15 on D2 day, Figure 4A). In addition, High caffeine consumers have a higher mean HR than the other two consumers groups, in the caffeine conditions (Figure 4B).

### 3.5 Biological parameters

The results of the statistical analyses are presented in the table below (Table 2).

**Table 2:** Results of mixed linear analyses for biological parameters.

Mixed linear model effects	IL-6	E-Selectin	MCP-1
Treatment (acute caffeine intake—TRT)	**5.21 (0.02)**	1.03 (0.08)	0.11 (0.94)
*F*(_1,34_, (p)			
Wake time (TSD)	**8.37 (0.01)**	**3.86 (0.01)**	**16.8 (0.01)**
*F*_5,170_, (p)			
Chronic caffeine consumption (CC)	**4.42 (0.02)**	0.27 (0.82)	0.12 (0.77)
*F*_5,170_, (p)			
TRT × TSD	1.63 (0.15)	0.57 (0.72)	1.52 (0.23)
*F*_5,170_, (p)			
TRT × CC	1.92 (0.07)	0.11 (0.98)	1.45 (0.43)
*F*_5,170_, (p)			
TSD × CC	1.21 (0.61)	**2.26 (0.02)**	1.58 (0.22)
*F*_5,170_, (p)			
TRT × TSD × CC	1.39 (0.19)	1.26 (0.51)	2.12 (0.06)
*F*_5,170_, (p)			

Bold values are significant values.

**Figure 5:**
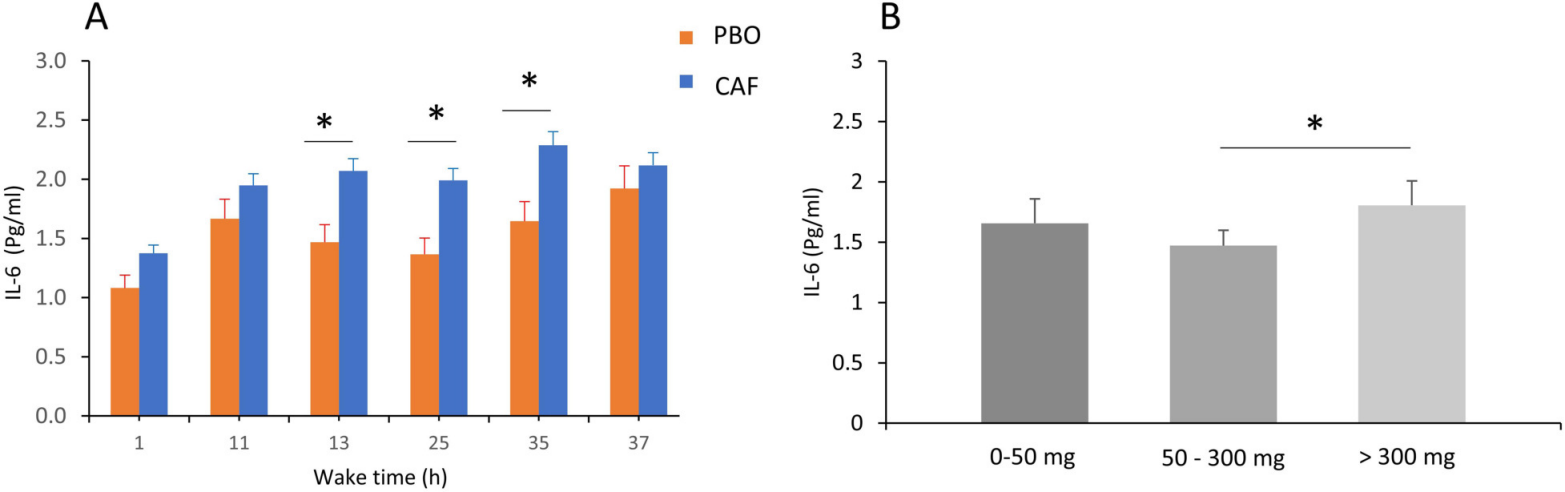
Plasma IL-6 concentrations (in pg/mL) in the acute caffeine (CAF) treatment compared to placebo (PBO) during total sleep deprivation (37 h of continuous wakefulness) **(A)**, and according to chronic caffeine consumption groups [<50 mg/day: low consumer group (*n* = 11); 50–300 mg/day: moderate consumer group (*n* = 12); >300 mg/day: high consumer group, *n* = 18] **(B)**, *p < 0.05, between PBO and CAF conditions and between high and moderate caffeine consumption groups.

Total sleep deprivation has significant effects on IL-6 and on all biological parameters (*p* < 0.01, Table 2). In addition, for IL-6 concentration there are acute caffeine intake and chronic caffeine consumption effects (*p* = 0.02 respectively), without significant interactions. The IL-6 concentration was higher in the caffeine condition compared with placebo, and the maximum increase during TSD was at 35 h of continuous wakefulness (Figure 5A). The IL-6 concentration was also significantly higher in high caffeine consumers (>300 mg/d) compared to moderate consumers (*p* < 0.05, Figure 5B).

**Figure 6:**
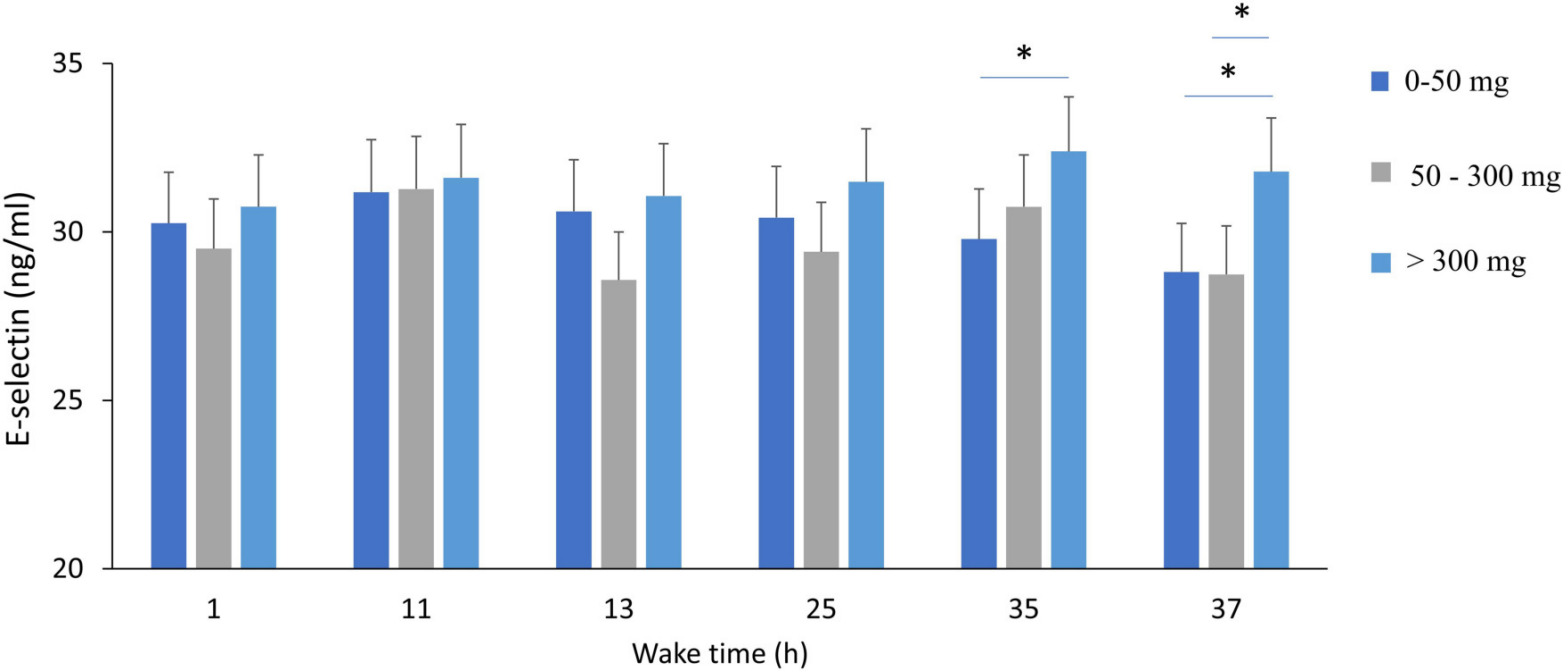
Plasma E-selectin concentrations during total sleep deprivation (37 h of continuous wakefulness), and according to chronic caffeine consumption groups [<50 mg/day: low consumer group (*n* = 11); 50–300 mg/day: moderate consumer group (*n* = 12); >300 mg/day: high consumer group, *n* = 18]. *p < 0.05, between caffeine consumption groups.

A significant effect of wake time (TSD) on plasma E-selectin concentrations is found (*p* = 0.01), with no effects of acute caffeine treatment or chronic caffeine consumption, but with significant interaction between TSD and chronic caffeine consumption (*p* = 0.02) (Table 2). The *post-hoc* analysis showed that high caffeine consumers have higher e-selectin concentrations at 35 and 37 h of continuous awakening, compared to low caffeine consumers. (Figure 6).

Only the wake time has a significant effect on MCP-1 plasma concentrations (*p* = 0.01), with no significant effect of acute caffeine treatment or chronic caffeine consumption (Table 2). The *post-hoc* analysis showed higher concentrations at 25 h compared to 1 h of awakening (338.3 ± 107.9 g/mL vs. 297.5 ± 97.5 pg/mL, *p* < 0.05).

### 3.6 Endothelial function measured at 14:00 on D1 and D2 days

The results of statistical analyses are presented in the table below (Table 3).

**Table 3:** Results of mixed linear analyses for local acetylcholine, heat and naCl application.

Mixed linear model effects	Acetylcholine (ACh)	NaCl	Heat
Treatment (acute caffeine intake—TRT)	0.01 (0.90)	0.02 (0.33)	1.15 (0.45)
*F*_1,76_, (p)			
Wake time (TSD)	**32.10 (0.001)**	**15.77 (0.001)**	**50.78 (0.001)**
*F*_2,59_, (p)			
Chronic caffeine consumption (CC)	**2.26 (0.02)**	1.16 (0.47)	1.15 (0.61)
*F*_14,28_, (p)			
TRT × TSD	0.13 (0.63)	0.67 (0.93)	0.90 (0.37)
*F*_11,76_, (p)			
TRT × CC	0.67 (0.45)	1.01 (0.43)	1.57 (0.13)
*F*_11,76_, (p)			
TSD × CC	2.22 (0.02)	0.97 (0.82)	1.21 (0.69)
*F*_11,76_, (p)			
TRT × TSD × CC	00.52 (0.66)	0.17 (0.95)	0.62 (0.75)
*F*_11,76_, (p)			

Bold values are significant values.

**Figure 7:**
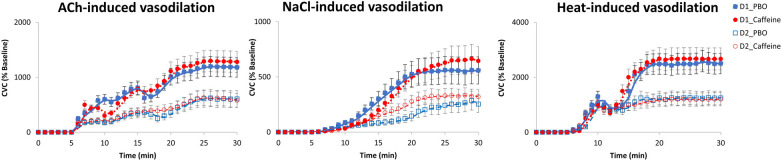
Cutaneous vascular conductance (CVC) (expressed as % of baseline  =  percentage of baseline) after local acetylcholine (ACh), naCl and heat applications, expressed before (D1 day) and after (D2 day) sleep deprivation, according to treatment (PBO or caffeine). PBO, placebo; CAF, caffeine.

**Figure 8:**
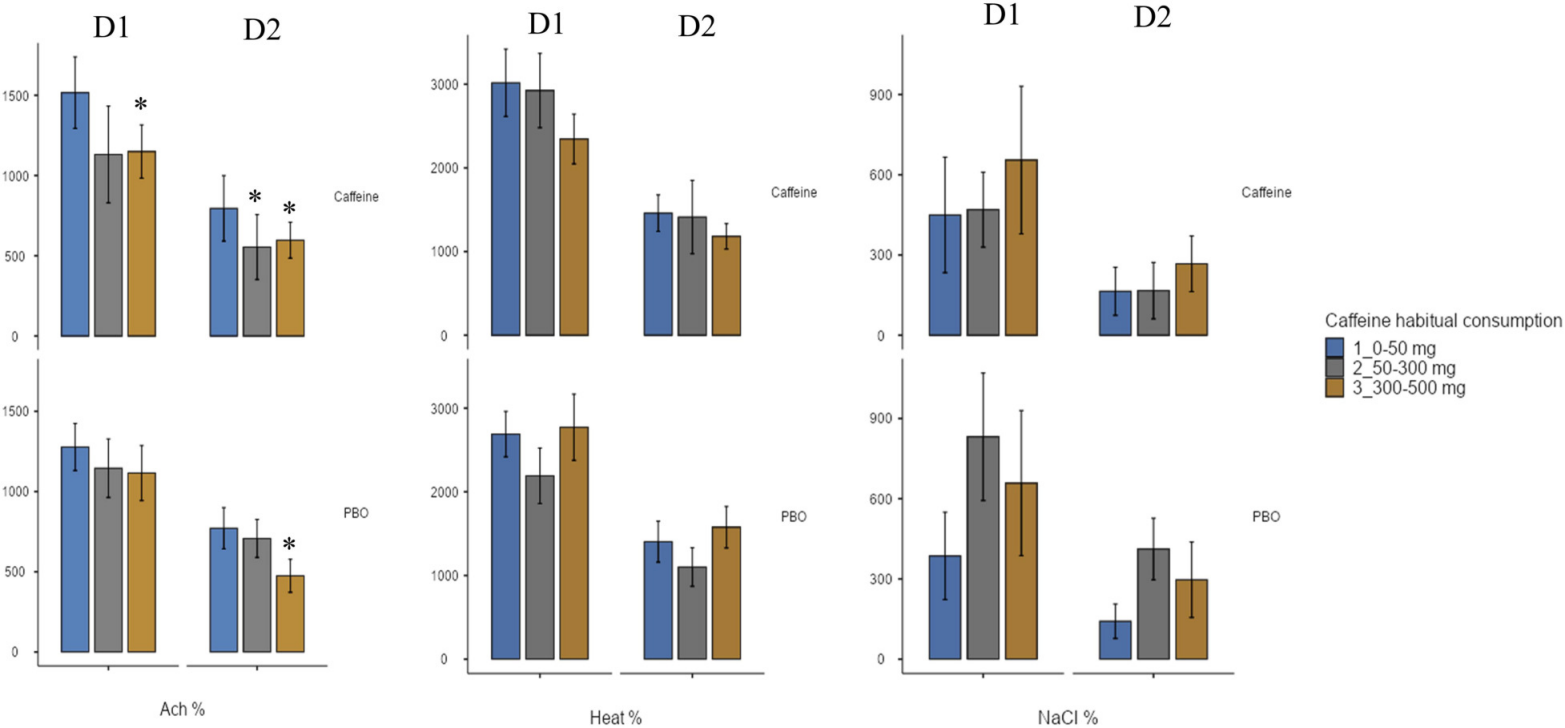
Maximum increase in cutaneous vascular conductance (CVC) (expressed as a % of baseline) after local application of acetylcholine (ACh), naCl, or heat, before (D1 day) and after (D2 day) sleep deprivation, according to treatment (PBO or caffeine), and to chronic caffeine consumtion groups (<50 mg/day: low consumer group; 50–300 mg/day: moderate consumer group; >300 mg/day: high consumer group). * difference versus low consumers (<50 mg/day).

A significant effect of wake time (TSD) is observed on the vascular response to local application of ACh, NaCl and Heat, without significant effect of acute caffeine treatment (Table 3). For ACh application, there are lower CVC after TSD compared with before (D2 vs. D1) both for placebo and caffeine condition (Figure 7), and we also observed an effect of chronic caffeine consumption (*p* < 0.02). High caffeine consumers have a lower maximal CVC (CVCpeak) response to ACh compared to low consumers after TSD (i.e., at D2 day), in the placebo or caffeine condition (Figure 8). Moderate caffeine consumers have a lower CVC response to ACh compared to low consumers after TSD (i.e., at D2 day), in the caffeine condition only (Figure 8). In addition, in the caffeine condition only, we observed a lower vascular reactivity to ACh in high caffeine consumers before TSD (i.e., at the D1 day) (Figure 8). We also found a linear relationship between CVC response to ACh and chronic caffeine consumption for both placebo and caffeine conditions during the D1 day (*p* = 0.02 respectively), while it is present only in the placebo condition during the D2 day (*p* = 0.02).

### 3.7 Correlations analysis

Chronic caffeine consumption was in significant positive correlation with SAP and MAP, whether considered specifically on D1 or D2, or as percentage change between the two days (D2-D1/D1) (*p* = 0.01 and *p* = 0.002, respectively). It also was in negative correlation with ACh-induced vasodilatation when considered either at D1 or D2 day specifically, or the percentage change between the two days (D2-D1)/D1) (*r* = −0.36, *p* = 0.001, for the percentage change between D2 and D1 days*; r* = −0.27, *p* = 0.01, for D1*;* and *r* = −0.25, *p* = 0.01, for D2). In addition, the IL-6 concentration was in negative correlation with ACh-induced vasodilatation when considering its percentage change between the two days (D2-D1)/D1) (*r* = −0.31, *p* = 0.01) i.e., when the subject was facing total sleep deprivation.

## 4 Discussion

The primary of this study was to evaluate the effect of acute caffeine intake on cardiovascular responses, i.e., arterial blood pressures, heart rate, vascular reactivity and related biomarkers, during a period of total sleep deprivation (TSD) in healthy subjects, taking into account their chronic caffeine consumption. To stay as close as possible to lifestyle habits, we chose to conduct this study in subjects who were not weaned off caffeine before the protocol. We observed that acute caffeine intake inflates the TSD-related increase in arterial blood pressures (systolic and diastolic), as well as blood IL-6 concentrations, the pro-inflammatory cytokine, but does not affect TSD-related ACh-induced vascular dysfunction. We also observed that high chronic caffeine consumption induced higher systolic blood pressure and interacted with TSD-related ACh-induced vascular dysfunction and e-selectin response.

Coffee and other caffeinated beverages are widely consumed daily. Therefore, it is important to define the potential risks and benefits associated with chronic caffeine consumption to better inform healthcare professionals and the public (19). The acute pressor effect of coffee and caffeine is well documented, but tolerance can develop rapidly with repeated use (18). In their 2005 meta-analysis of 16 randomized controlled trials, Noordzij et al. reported an increase in both systolic and diastolic blood pressure with regular coffee or caffeine consumption, with elevations greater in caffeine trials than in coffee trials, and the increase in systolic blood pressure greater in high-dose caffeine trials (≥410 mg/day) than in lower-dose caffeine trials. Effects on heart rate were negligible and not statistically significant (39). One year later, Umemura et al. (40) described significant increases in peripheral systolic and diastolic pressures and unaltered heart rate one hour after oral administration of 300 mg of caffeine in young nonhabitual caffeine consumers. In comparison, Waring et al. (41) described that 300 mg of caffeine administration increased central (aortic) systolic and diastolic blood pressures, but had no effects on peripheral blood pressure, in healthy subjects with regular moderate coffee consumption. In this study, the acute caffeine caused a significant decrease in heart rate. More recently, a meta-analysis of 11 randomized controlled trials showed an overall blood pressure elevation for short-term (within four weeks) caffeinated beverages intake, with low-dose caffeine intakes (≤245 mg) raising only SBP and high-dose intake (>245 mg), increasing both SBP and DBP (42). Furthermore, acute ingestion of coffee (containing 80 mg of caffeine) was shown to result in a significant decrease in endothelium-dependent flow-mediated dilation (FMD) of the brachial artery 30 and 60 min after ingestion (43). Finally, chronic high coffee consumption (>450 mL per day, i.e > ∼300 mg caffeine per day) has been shown to be associated with higher aortic stiffness and wave reflections in healthy subjects, even after controlling for various confounding factors (such as age, subjects aged around 41 years, sex, smoking habits, body mass index,…), these factors explaining the higher peripheral systolic and diastolic pressures (44). To our knowledge, there are no data regarding the effects of acute caffeine intake on blood pressure during prolonged wakefulness, taking into account chronic caffeine consumption.

In our study, we observed significant main effects of acute treatment (caffeine or placebo) and wake time on systolic (SAP) and diastolic (DAP) blood pressures with higher values in the caffeine condition compared to the placebo condition, particularly between 2 and 14 h of continuous wakefulness, i.e., not before being deprived of sleep, and from 45 min of caffeine ingestion. This is observed in healthy subjects not previously weaned in caffeine. This result confirms the recent meta-analysis of Xu et al. (42) as well as the study by Umemura et al. (40) who describe increased peripheral blood pressures 1 h after ingestion of 300 mg of caffeine in young healthy subjects who do not consume caffeine every day (i.e., nonhabitual consumers), and with only 24 h of abstinence before entering the protocol. We also confirmed the latter study showing that acute caffeine intake did not alter heart rate (40). Furthermore, continuous wakefulness increased blood pressures and heart rate in both treatment conditions, after accumulating 26 h of wakefulness, as we have previously described, for SAP particularly and also for heart rate (7). In our study, subjects self-measured their blood pressure at three consecutive times, being previously formed and with explanatory papers displayed in front of them at the time of the blood pressure measurement, in order to respect health service recommendation currently in use, and to avoid a white coat effect, which could have induced false high blood pressure measurements (30).

With regard to the influence of the chronic caffeine consumption, our results indicated a main effect on SAP only, with higher SAP values in high consumers (>300 mg/day, or about 3 cups of coffee per day) than in low caffeine consumers, even before being sleep deprived, and whether they belonged to the caffeine or the placebo condition. This latter observation confirms the literature on the blood pressure effects of regular (or chronic) coffee/caffeine consumption suggesting that high daily consumption may be an additional risk factor in the development of hypertension (18, 39). Statistical analysis also showed a main effect on the heart rate with high caffeine consumers having higher values than low consumers, even before being sleep deprived, in both the placebo and caffeine conditions. This result was partially observed in the meta-analysis by Noordzij et al. (39), as the authors instead described a non-significant increase in HR with chronic coffee or caffeine consumption. In our study, the inclusion of chronic caffeine consumption in the statistical analysis provides original elements regarding its interaction with acute caffeine intake on the cardiovascular and inflammatory response of healthy subjects in a degraded situation of total sleep deprivation.

Regarding the three inflammatory biomarkers related to the endothelial function, E-selectin, MCP-1 and the pro-inflammatory cytokine IL-6, we confirmed some of our previous results and others from the literature with a main effect of sleep deprivation (7, 38, 45). Main effects of acute treatment (caffeine intake) and chronic caffeine consumption were observed only on IL-6 levels, and a significant interaction was present between chronic caffeine consumption and sleep deprivation for E-selectin. There is a higher IL-6 level after acute caffeine intake as previously described (after an acute intake of 6 mg/kg of body mass, i.e., similar to that in our study) (37), and moderate chronic caffeine consumers (50–300 mg) have lower IL-6 levels than high consumers, which could be in favor of an anti-inflammatory effect described in the literature but rather for coffee and not for caffeine consumption (46). Post-analysis of E-selectin response to sleep deprivation according to chronic caffeine consumption shows higher levels after accumulating 35 and 37 h of wakefulness in the high caffeine consumers compared to low and moderate consumers. As E-selectin is one of the traditional cardiovascular risk factors (47), we can suggest that high caffeine consumption may exacerbate the deleterious effect of sleep deprivation on cardiovascular risks. E-selectin is involved in leukocyte rolling on the endothelium and platelet-leukocyte interaction, as it is expressed in activated endothelial cells and acts as an adhesive reagent, and upon activation it is released into the circulation.

With respect to the vascular reactivity response (i.e., cutaneous cardiovascular conductance, CVC, expressed in % of baseline) to ACh during total sleep deprivation with acute caffeine or placebo intake, our results confirmed its decrease after sleep deprivation as previously described (7, 38), and additionally showed no significant difference between caffeine and placebo condition. We also confirmed the sleep deprivation-induced a CVC decreased response after heat (38) and NaCl applications, and we additionally showed no significant effect of acute caffeine intake nor chronic caffeine consumption. Local Ach application induces cutaneous vasodilation via the endothelium-dependent release of vasoactive substances such as nitric oxide (NO), and heat application induces a cutaneous vasodilation that initially depends on the axonal reflex, and subsequently depends on local NO production. While no effect of acute caffeine is observed for ACh application, a main effect of chronic caffeine consumption is observed with significant interaction with sleep deprivation. In the literature, there are few studies examining the effects of acute caffeine intake on vascular function in young healthy individuals, and no studies to our knowledge under conditions of total sleep deprivation. The result for ACh application can be compared to that of Duffy et al. (48) who evidenced no effect of 200 mg of caffeine ingestion at 2 h post ingestion on the FMD (endothelium-dependent flow-mediated dilatation) of the brachial artery, and to that of Papamichael et al. (43) who evidenced an acute effect of 80 mg of caffeine ingestion with a decline of FMD at 30- and 60-min post ingestion. However, our result contrast with the study of Umemura et al. (40) showing that the FMB response to ACh is augmented one hour after acute administration of caffeine (300 mg) (with increased systolic and diastolic blood pressures and no change of heart rate as indicated above) in healthy young nonhabitual caffeine consumers. In our study, the lack of acute effect of caffeine is also not surprising because the FMD was measured at 14:00, which is much too far from the caffeine dose (08:30). This study was part of a larger laboratory experimental protocol investigating the effects of caffeine intake during total sleep deprivation on cognitive responses, with the primary endpoint here being blood pressure measured every two or six hours. For us, it was reasonable to measure vascular reactivity only twice, before and after 24 h of sleep deprivation, because the protocol is invasive and cumbersome to set up and to be accepted by the subjects (and the ethics committee), and also to be able to compare with our previous studies (7, 38).

When we considered the effect of chronic caffeine consumption and ACh induced CVC, high caffeine consumers presented lower values at the maximal CVC level compared to low consumers, at the D2 day i.e., after sleep deprivation and in the two acute treatment conditions, caffeine and placebo. This was also observed at the D1 day before sleep deprivation in the caffeine condition. High caffeine consumers therefore exhibit, before and after sleep deprivation, lower ACh-induced vasodilatation and higher IL-6 levels, and higher sleep deprivation-related E-selectin levels, which together constitute a cardiovascular risk factor. Correlation analysis highlights the relationship between high chronic caffeine consumption and low ACh-induced vasodilation and high SAP on one side, and between high IL6 concentration and the sleep deprivation related ACh-induced vasodilation on the other side, linking high caffeine consumption and the inflammation that would be related to it, to cardiovascular risk in healthy subjects. In any case, the lower vascular reactivity to ACh in high caffeine consumers with higher E-selectin levels could contribute to higher peripheral SAP as we observed. Indeed, one of the functions of vascular reactivity is the regulation of peripheral resistance, particularly during a situation of increased blood pressure. The endothelium regulates vascular tone, platelet activity, leukocyte adhesion, and angiogenesis by producing nitric oxide and other regulatory factors, and eendothelial dysfunction is a pathological condition, mainly characterized by an imbalance between substances with vasodilating and vasoconstricting characteristics (49). Regarding caffeine, its vascular effects (vasoconstriction or vasodilation) may vary depending on the amount of caffeine absorbed (50). Caffeine has various pharmacological actions, including: (1) adenosine receptor antagonist action, (2) inhibition of phosphodiesterase (PDE), (3) increase in intracellular calcium concentration, (4) production of endothelium-derived hyperpolarizing factor (EDHF), (5) decrease in oxidative stress, and (6) enhancement of endothelial nitric synthase (eNOS) expression for the regulation of vascular function (51). Some researchers have hypothesized that caffeine is a vasoconstrictor substance (52, 53), which would be confirmed by the increase in blood pressures that we observed after acute caffeine intake. Additionally, adenosine is well known to induce vasodilation, thus antagonization of its receptors by caffeine, could induce vasoconstriction. In our study, the lack of acute caffeine effects on CVC could be due to its half-life pharmacokinetic which is generally between 4 and 6 h, and the lower CVC induced by Ach in high caffeine consumers will therefore be due to limitation of vasodilation and promotion of an increased sympathetic activity, highlighted by higher heart rate (54). Our double-blind caffeine vs. placebo study provides new insights into the adverse effects of chronic caffeine consumption on vascular reactivity, even if the number of subjects remains limited.

### 4.6 Limitations

Our work has a number of limitations, dominated by the size of our sample and therefore interindividual variability. However, the duration of the experiment and the constraints imposed on subjects in controlled laboratory conditions constitute an obstacle to a larger sample. Furthermore, it was not possible to measure heart rate variability using the method employed, which consisted of taking three successive self-measurements of blood pressure and heart rate. In future studies, it would be relevant to confirm our results and supplement them with measurements of autonomic nervous system activity. It would be additionally interesting to study the cardiovascular effects of caffeine during sleep deprivation on a larger and different population, for example during professional practice (hospital care, operational activity for the military, etc.). In addition, other parameters such as age or sex or genetics cause inter-individual variability in physiological response to sleep deprivation and the effects of caffeine consumption (26). Considering genetic background would make it possible to identify the subjects most at risk of vascular responses. Finally, it would also have been relevant to assess the effects of recovery night on systolic and diastolic blood pressure, heart rate, inflammatory, immunologic parameters and vascular reactivity, depending on the category of caffeine consumers.

## 5 Conclusions

Chronic caffeine consumption, beyond one coffee a day (about >50 mg/day), has effects on the cardiovascular system during total sleep deprivation. Heavy consumers of caffeine (>300 mg/day, or about 3 espresso coffees per day) have higher blood pressure than low consumers, even before sleep deprivation. They also exhibit an alteration of endothelial function and thus an increase in cardiovascular risk over the long term. The physiological response to acute caffeine intake, however, remains subject to significant inter-individual variability (chronic caffeine consumption as we demonstrate in this study, genetics, sex, age, fitness status, etc.). Therefore, the use of caffeine by workers with sleep debt should be regulated. The daily consumption of caffeine should be reduced (<300 mg/day) by favoring acute intake when signs of drowsiness appear. Caffeine should be avoided 4 to 6 h before bedtime to avoid disrupting the night's recovery (55). Response to caffeine intake should be evaluated individually before use during a period of sleep deprivation.

## Data Availability

The raw data supporting the conclusions of this article will be made available by the authors, without undue reservation.
